# 

**Published:** 1893-10

**Authors:** 


### CONTENTS:

Article I.—Dentistry: Past, Present and Future. By A. C. Cogswell, D. D. S. - - - - - - - 241
" II.—Conservative Treatment of the Dental Pulp. - - 251
" III.—Twenty-Fourth Annual Session of the Alabama Dental Association. - - - - - - - 266
" IV.—Cocaine in Surgery. By R. H. Cowan. - - - 274
" V.—Prosthetic Dentistry. By Dr. Haskell. - - - 276

### EDITORIAL.

Changes in the University of Maryland. - - - - - 281
Education. - - - - - - - - - 281

### MONTHLY SUMMARY.

A Simple Holdfast for Porcelain Inlays.. - - - - - 282

The Use of Cocain as an Anæsthetic. - - . - - 282

My Method. - - - - - - - - - 283

Nitrate of Silver. - - - - - - - - 284

Remarkable Surgical Operation. - - - - - - 285

Lysol. - - - - - - - - - - 286

Women Dentists. - - - - - - - - 287

How I Treated an Abscess. - - - - - - - 288

Recent Experiments. - - - - - - - 288

